# Nutritional Status and Circulating Levels of Fat-Soluble Vitamins in Cystic Fibrosis Patients: A Cohort Study and Evaluation of the Effect of CFTR Modulators

**DOI:** 10.3390/children10020252

**Published:** 2023-01-30

**Authors:** Michela Francalanci, Vito Terlizzi, Cristina Fevola, Giulia Di Rosa, Valentina Pierattini, Elena Roselli, Paolo Bonomi, Maria Chiara Cavicchi, Valeria Galici, Anna Silvia Neri, Chiara Bianchimani, Silvia Campana, Daniela Dolce, Novella Ravenni, Erica Camera, Tommaso Orioli, Giovanni Taccetti

**Affiliations:** 1 Meyer Children’s Hospital IRCCS, Cystic Fibrosis Regional Reference Centre, Department of Paediatric Medicine, 50139 Florence, Italy; 2 Meyer Children’s Hospital IRCCS, Professional Dietetics, 50139 Florence, Italy; 3 Freelance Statistician, 20146 Milan, Italy

**Keywords:** cystic fibrosis, malnutrition, CFTR modulators

## Abstract

Background: Improved therapy in CF has led to an overall improvement in nutritional status. The objectives of our study are: to cross-sectionally assess nutritional status and serum levels of fat-soluble vitamins; to retrospectively evaluate the efficacy of modulators on nutritional status and fat-soluble vitamin levels. Methods: In patients younger than 2 years of age, we evaluated growth, in patients aged 2–18 years, we assessed BMI z-scores, and in adults, we assessed absolute BMI values. Levels of 25(OH)D, vitamins A, and E were measured. Results: A cross-sectional analysis was conducted on 318 patients, 109 (34.3%) with pancreatic sufficiency. Only three patients were under 2 years old. In 135 patients aged 2–18 years, the median BMI z-score was 0.11, and 5 (3.7%) patients had malnutrition (z-score ≤ 2SD). In 180 adults, the median BMI was 21.8 kg/m^2^. Overall, 15 (13.7%) males (M) and 18 (25.3%) females (F) were underweight (18 < BMI > 20); 3 (2.7%) M and 5 (7.0%) F had a BMI < 18. Suboptimal 25(OH)D levels were found in patients with pancreatic insufficiency. The prevalence of deficiency of vitamins A and E is low. After one year of treatment with modulators, the increase in BMI was more consistent (M: 1.58 ± 1.25 kg/m^2^ F: 1.77 ± 1.21 kg/m^2^) in elexacaftor/tezacaftor/ivacaftor (ETI)-treated patients compared with other modulators, with a significant increase in levels of all fat-soluble vitamins. Conclusions: Malnutrition is present in a limited number of subjects. The prevalence of subjects with suboptimal 25(OH)D levels is high. ETI showed a beneficial effect on nutritional status and circulating levels of fat-soluble vitamins.

## 1. Introduction

Nutritional status in cystic fibrosis (CF) patients should be considered an important prognostic factor and is a key parameter in the overall assessment of the impact of the disease [1,2,3,4,5,6,7,8]. Malnutrition and growth retardation in CF are the result of a negative energy balance secondary to maldigestion and malabsorption [1,8,9,10,11,12,13,14,15,16,17,18]. In the past, the wasting of the body contributed to the progressive evolution of the disease; subsequently, thanks to increased attention to nutritional status and increasingly innovative therapies [1,4,5,6,7,8], malabsorption has been limited, and the prognosis has consequently improved. Because of their importance, data on nutritional status are collected in major CF Registries and are carefully evaluated [4,5,6].

Innovative therapies with modulators of CF transmembrane conductance regulator (CFTR) protein have recently been introduced into clinical practice with the prospect of further improvement in patients’ clinical condition and nutritional status.

The aims of the present study are:

- To cross-sectionally evaluate CF patients’ nutritional status and serum levels of fat-soluble vitamins.
- To retrospectively evaluate the efficacy of treatment with CFTR protein modulators in terms of changes in nutritional status and circulating fat-soluble vitamin levels.

The effect of modulator therapy on circulating levels of fat-soluble vitamins has only been described occasionally [19].

## 2. Materials and Methods

Anthropometric data of CF patients in the quarterly follow-up, in December 2020, at the CF Centre of Florence were collected and evaluated [4]. This study was approved by the local ethics committee. CF diagnosis fulfilled international criteria [7,8]. Patients were considered pancreatic insufficient based on elevated levels of fat in stools [4].

In all patients, treated according to the standard of care, we assessed exocrine pancreas function, gave guidance regarding pancreatic enzyme replacement therapy if necessary, planned a diet with appropriate fat intake offered behavioural therapy to educate the patient during mealtimes and suggested sodium chloride supplementation when necessary.

In all patients with pancreatic insufficiency we suggested fat-soluble vitamin supplementation with dosage in accordance with the standard of care [7].

Exclusion criteria were:

- Subjects who had undergone organ transplantation;
- Subjects diagnosed with CF Related Disorders;
- Subjects categorized as screening positive with inconclusive diagnosis (CFSPID).

For subjects younger than 2 years of age, weight, length, and relative percentiles were calculated (reference values: WHO) [4].

In subjects aged 2–18 years, weight and height were measured, and BMI was expressed in terms of z-scores and percentiles (reference values issued by the Center for Disease Control, USA) [4,5,6].

According to their BMI z-scores, patients were stratified into 4 categories:

- ≥0 SDS;
- <0 SDS, ≥−1 SDS;
- <−1 SDS, ≥−2SDS;
- <−2 SDS.

For adults (>18 years), the absolute values of BMI kg/m^2^ and percentiles were calculated.

Based on BMI values, nutritional status was defined [4] as in Table 1:

The distribution of BMI values (mean, 25th percentile, median, and 75th percentile) in our study population was analysed and compared with data from the European Cystic Fibrosis Registry (ECFS) [4].

Circulating levels of 25-hydroxyvitamin D [25(OH)D], vitamins A, and E [20,21,22,23,24,25] were assessed at the annual review in all follow-up patients and were used as biomarkers of nutritional status.

25(OH)D in serum was measured by chemiluminescent immunoassay technology [25], and Vitamins A and E were determined by HPLC-UV [19,20,21,22,23,24].

The reference values [7,8,9,10,11,12,13,14,15,16,17,18,19,20,21,22] were:

- 25(OH)D: normal values ≥ 30 ng/mL; vitamin insufficiency 10–29 ng/mL; vitamin deficiency < 10 ng/mL; toxicity > 100 ng/mL [22];
- Vitamin A: normal values: 30–70 mcg/dL;
- Vitamin E: normal values: 500–2000 mcg/dL.

In a subset of patients who had been treated with CFTR protein modulators for at least one year, the treatment’s effect on nutritional status and circulating fat-soluble vitamin levels was retrospectively evaluated. The duration of follow-up regarding nutritional status in patients treated with modulators depended on the different timing of the introduction of the various specialties on the market in Italy.

### Statistical Analysis

All collected values were entered into an electronic database. Descriptive statistics for quantitative variables were performed using normal distribution tests. Comparisons between independent samples were performed using Student’s *t-*test for the equality of the means.

The effect of CFTR modulators on serum levels of fat-soluble vitamins was evaluated using a *t*-test for paired data.

The level of statistical significance was expressed as a *p*-value and it was considered statistically significant if *p* was <0.05.

## 3. Results

A total of 83 subjects (32 with lung transplantation, 28 who did not provide consent, 22 CFSPID subjects in follow-up, and 1 with CF Related Disorders) were excluded.

### 3.1. Patients’ Demography

Nutritional status was assessed in 318 (79.3%) of 401 patients (mean age of 24.5 years; median of 22 years; range of 2–67) in follow-up to December 2020. Overall, 191 (60%) patients out of 318 had been diagnosed by newborn screening [26].

Patients were divided by age group into 3 categories [4]:

- <2 years: 3 patients (1 male and 2 females), mean age ± SD 1.67 ± 0.58 years;
- 2–18 years: 135 patients (69 males and 66 females) mean age ± SD 10.70 ± 3.90 years;
- >18 years: 180 patients (109 males and 71 females) mean age ± SD 34.85 years; ±11.80.

### 3.2. Nutritional Status

In the age group of 0–2 years (only 3 patients), weight and length percentiles were within the normal range. In one patient diagnosed at birth with a severe picture of meconium ileus, the weight and length percentiles were lower (weight: 0.4th centile; length: 13th centile).

In the population aged 2–18 years, 3 (2.2%) of 135 patients had a height-for-age z-score value < 2 SD (stunting).

Table 2 shows the BMI z-score classes in this age group.

Regarding the BMI z-scores assessment, in our case study, the mean is 0.014, the median 0.11, the 25th percentile −0.62, and the 75th percentile 0.71.

When compared to CDC percentiles [6], the median found in our population corresponds to the 55.6th percentile, the 25th percentile to 26.7, and the 75th percentile to 76.5.

In adults, the median of absolute BMI values is 21.81 kg/m^2^, the mean is 22.14 kg/m^2^, the 25th percentile is 20.16 kg/m^2^ and the 75th percentile is 23.76 kg/m^2^. The median of BMI values for males is 22.6 kg/m^2^, the 25th percentile is 20.6 kg/m^2^, and the 75th percentile is 24.6 kg/m^2^. In females, the median is 21.1 kg/m^2^, the 25th percentile is 19.7 kg/m^2^, and the 75th percentile is 22.2 kg/m^2^. Table 3 describes the absolute BMI values by classes in patients > 18 years old.

A total of 15 (13.8%) males and 18 (25.4%) females were underweight; 3 (2.7%) males and 5 (7.0%) females were malnourished.

We found 51 (16.2%) patients to be overweight (25 ≤ BMI < 30) and 2 (0.6%) obese (BMI ≥ 30) [27,28].

The mean ± SD value of BMI was 21.7 ± 2.6 in 209 (65.7%) of 318 adult subjects with pancreatic insufficiency and 23.2 ± 3.1 in the 74 (23.3%) adults with pancreatic sufficiency. The difference between the two groups was statistically significant (*p* value = 0.001).

### 3.3. Fat-Soluble Vitamin Levels

Serum levels of 25(OH)D were assessed in 283 (89%) of 318 patients considering the period April–September as having the highest sun exposure and October–March as having low sun exposure. The patients were divided into patients with pancreatic exocrine sufficiency and insufficiency [4,29,30,31], and serum vitamin concentration values were compared.

Patients with pancreatic sufficiency in the spring–summer months showed mean ± SD values of 25(OH)D of 32.78 ± 10.14 ng/mL. Patients with vitamin insufficiency totalled 107 (38%). In the fall–winter months, the mean ± SD values of 25(OH)D were 27.16 ± 10.9 ng/mL. There were 167 (59%) patients with vitamin insufficiency [22]. No patients with preserved pancreatic function had values < 10 ng/mL.

Considering patients with pancreatic insufficiency, mean ± SD values of 25(OH)D in the spring–summer months were 30.13 ± 11.2 ng/mL. Patients with vitamin insufficiency totalled 144 (51%); patients with complete vitamin deficiency totalled 8 (3%). In the autumn–winter months, the mean ± SD value of serum 25(OH)D was 28.0 ng/mL ± 12.6. Patients with vitamin insufficiency totalled 184 (65%), and patients with complete vitamin deficiency totalled 6 (2%).

Vitamin A levels were tested in 243 (76.4%) patients. Mean ± SD levels were 42.6 ± 12.3 mcg/dL; 30 (12.3%) patients had vitamin insufficiency (<30 mcg/dL), and 5 (2%), including 2 on modulator treatment, had hypervitaminosis (>70 mcg/dL) without any symptoms [19,24].

Vitamin E levels were measured in 250 (78.6%) patients. Mean ± SD levels were 1106 ± 336 mcg/dL; 4 (1.6%) patients had vitamin insufficiency (<500 mcg/dL) and 4 more (1.6%) had hypervitaminosis (>2000 mcg/dL) without any symptomatology [19,20,21,22,23,24].

### 3.4. Impact of CFTR Modulators

As of December 2020, 80 (25.1%) of 318 follow-up patients had been on modulators for at least 1 year. Patients were divided by modulator type [32,33,34,35,36,37,38,39,40,41,42,43,44,45,46,47,48,49,50,51,52,53,54,55], age (2–18 years and ≥18 years) and gender. There were 9 patients on Ivacaftor (Iva), 42 patients on Lumacaftor/Ivacaftor (LumIva), and 29 patients on Elexacaftor/Tezacaftor/Ivacaftor (ETI).

**Figure 1:**
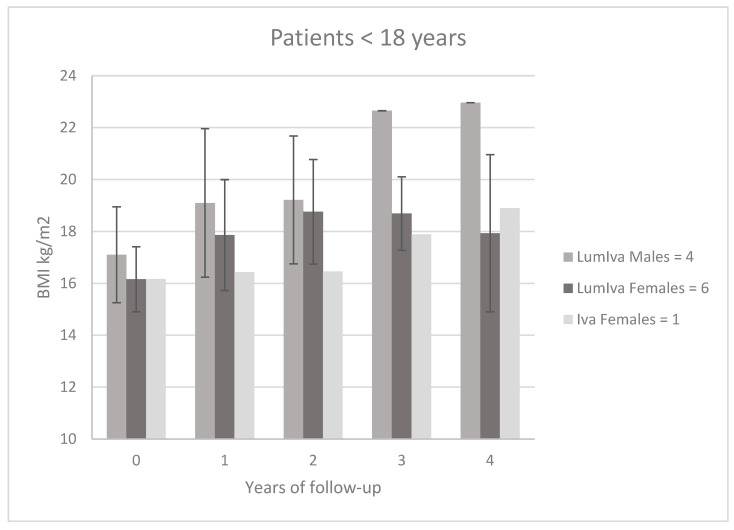
BMI trend (mean ± SD) in 4 males and 6 females aged between 2–18 years treated with LumIva and one female treated with Iva.

Figure 1 shows the average BMI trend in 4 males and 6 females aged between 2 and 18 years treated with LumIva and 1 female treated with Iva. The follow-up period for these patients was four years.

**Figure 2:**
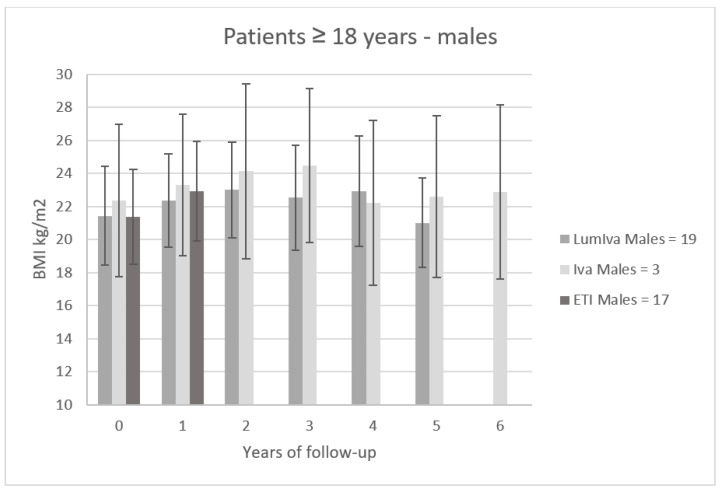
BMI trend (mean ± SD) in 39 male patients aged ≥ 18 years treated with modulators (Iva N = 3, LumIva N = 19, and ETI N = 17).

Figure 2 shows the trend of BMI (mean ± SD) in 39 male patients aged ≥ 18 years treated with modulators (IVA N = 3, LumIva N = 19 and ETI N = 17). The follow-up period of these patients was 6 years for patients treated with Iva, 5 years for patients treated with LumIva and 1 year for patients treated with ETI. After 1 year of treatment, we compared the efficacy of the three modulators in terms of BMI. Male patients on LumIva therapy improved their BMI on average by 0.94 ± 0.92 kg/m^2^, patients on Iva therapy improved by 0.96 ± 0.44 kg/m^2^, and patients on ETI therapy improved by 1.58 ± 1.25 kg/m^2^.

**Figure 3:**
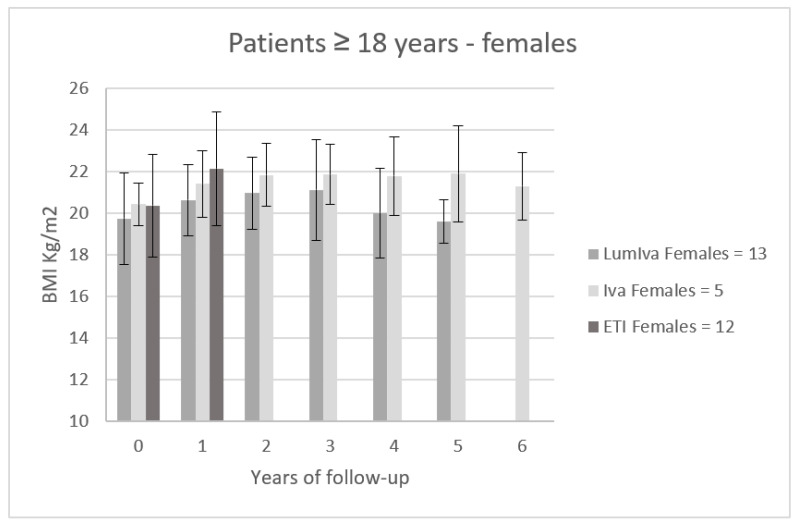
BMI trend (mean ± SD) in 30 female patients aged ≥ 18 years treated with modulators (IVA N = 5, LumIva N = 13 and ETI N = 12).

Figure 3 shows the trend of BMI (mean ± SD) in 30 female patients aged ≥ 18 years treated with modulators (IVA N = 5, LumIva N = 13 and ETI N = 12). The follow-up period of these patients was 6 years for patients treated with Iva, 5 years for patients treated with LumIva, and 1 year for patients treated with ETI. Within the time frame of the first year, females on LumIva therapy had an improvement of 0.88 ± 1.08 kg/m^2^, females on Iva therapy had an improvement of 0.99 ± 1.01 kg/m^2^, and females on ETI had an improvement of 1.77 ± 1.21 kg/m^2^.

Table 4 shows the mean ± SD values of fat-soluble vitamin levels detected in the serum of patients before and after modulator treatment. A statistically significant increase was observed in patients on Iva treatment with regard to Vitamin E levels (*p* = 0.036), and in patients on LumIva treatment with regard to Vitamin A (*p* = 0.008). A statistically significant increase in levels of all circulating fat-soluble vitamins was observed in subjects on ETI treatment.

## 4. Discussion

Nutritional status is a major prognostic factor in CF and influences multiple clinical variables, such as respiratory function (FEV_1_) and pulmonary exacerbations [1,5,13,14,15,16].

Malnutrition is associated with worse prognosis such as increased frequency of hospitalizations and increased comorbidities and mortality [1,5,13,14,15,16,17,18].

The ECFS Standards of Care recommend percentiles of weight and height similar to those of the normal population for children up to 2 years of age, a value above the 50th percentile of BMI for individuals up to 18 years of age, and a BMI above 20 kg/m^2^ for adults [4].

In Tuscany, the low birth rate and genetic counselling performed after newborn screening have reduced the incidence of CF over time [26]. In our case series, only three patients are between 0–2 years of age, and no statistical evaluation can be performed.

In patients between 2 and 18 years of age, we observed three subjects with height z-score values suggestive of stunting. The BMI z-score values by age in our case series are similar to the Italians patients in the European CF registry (2020) [4], showing a mean of −0.1, a 25th percentile of −0.7, a median of 0.0, and a 75th percentile of 0.7. Since the percentage of subjects with BMI z-scores < −2SDS is 4.35% for M and 3.03% for F in this age group, the percentage of subjects with malnutrition is limited, and there is no “gender gap”.

In our study, the adult BMI values are lower than those observed in the European Patient Registry [4], where the mean is 22.6 kg/m^2^, the 25th percentile 20.2, the median 22.1 and the 75th percentile 24.5, and the data are from the US Registry, where the median is 23.6 kg/m^2^. The reasons for these differences are difficult to find, but unlike the paediatric population, the adults’ nutritional status shows the existence of a “gender gap” in the CF population attending our Centre.

Exocrine pancreatic function is important in the course of the disease [16,17,29,30,31], as we observed a difference in BMI values between patients with pancreatic sufficiency and insufficiency.

An additional parameter indicative of malabsorption and closely related to pancreatic activity is the serum 25(OH)D level, which can be influenced by many factors [22]. The prevalence of subjects with suboptimal levels is higher in subjects with pancreatic insufficiency. We also found a difference between values in the spring–summer and fall–winter periods. It is now recommended that serum 25(OH)D concentration be checked annually, preferably at the end of winter, and that its level is maintained above 30 ng/mL [22]. Recent studies have shown that optimal vitamin D values are associated with better lung function and can promote recovery from pulmonary exacerbations [21,22]. In a small number of patients, although in the absence of symptomatology, we also observed vitamin A and E deficiency [20].

Anthropometric data and fat-soluble vitamin levels were retrospectively analysed to assess the role of CFTR modulators [32,33,34,35,36,37,38,39,40,41,42,43,44,45,46,47,48,49,50,51,52,53,54,55] in improving nutritional status.

In Ivacaftor-treated subjects, after the first year of therapy BMI improved by an average of 0.96 kg/m^2^. After 12 months of Ivacaftor, the average improvement in BMI from baseline in 108 patients > 20 years old therapy was 0.9 kg/m^2^ [39]. A retrospective study documented an increase in mean BMI value (from 21.2 ± 1.5 kg/m^2^ to 22.1 ± 1.9 kg/m^2^) in adults treated with Ivacaftor for one year. In other experiences evaluating a 6-month time period, an increase of 0.8 kg/m^2^ was observed [40].

Regarding LumIva, in an analysis conducted in a French population > 18 years old, an average increase in BMI of 0.5 kg/m^2^ was found after 12 months of treatment, with no significant increase in vitamin A and E levels [46]_._ In our case series, we observed that over multiple years of therapy, the effect of LumIva on the nutritional status of our patients, especially in female subjects, was reduced. In addition, patients showed no increase in serum 25(OH)D or vitamin E levels. The limited number of patients studied, the short length of the observation period, the variable course of the disease, and the absence of data regarding the compliance of patients, particularly with regard to the intake of vitamins and/or modulators, represent limitations of our study and do not allow us to draw any conclusions. Therefore, the reasons for these observations remain a matter of speculation.

Ridley et al. analysed a cohort of 116 patients aged 12 to 17 years and compared the efficacy of LumIva therapy with ETI [53]. After a 6-month treatment with dual therapy, the average BMI increased by 0.1 kg/m^2^, while with ETI, the average improvement was 1.04 kg/m^2^. The superiority of triple therapy over dual therapy in terms of effectiveness on improving nutritional status was also demonstrated by our data showing a more consistent improvement in patients treated with ETI.

A multicentre randomised double-blind phase 3 study evaluated the efficacy of triple therapy (ETI) after a 4-week time frame, finding a mean increase in BMI of 0.6 kg/m^2^.

Petersen et al. evaluated the efficacy of triple therapy with ETI over a mean time interval of 12.2 months and found an increase in BMI of 1.47 kg/m^2^ [52].

In terms of BMI increase, the literature analysis and our data showed substantial differences between cohorts of patients treated with different modulators [38,39,40,41,42,43,44,45,46,47,48,49,50,51,52,53,54,55]. The modulators’ efficacy on disease outcomes is different, and ETI is now considered a “highly effective modulator”, a term that emphasizes the high efficacy of this drug on improving pulmonary function and sweat chloride levels [55].

In conclusion, to date, a limited number of patients, especially in paediatric age subjects, have a poor nutritional status. Improvement of nutritional status in paediatric age is a well-known positive effect of newborn screening, a strategy which has long been performed in our Region. In our case series, however, it is still evident that there is a “gender gap” in the adult population, which was probably affected by a severe course of the disease in the “pre-modulator era”. Especially in adults, there is a need to maintain high attention to continuous and regular monitoring of nutritional parameters, with the adoption of appropriate interventions where necessary.

In agreement with data in the literature [19,20,21,22,23,24], the majority of patients have values in the range for vitamin A and vitamin E. In contrast, the prevalence of suboptimal levels of 25(OH)D is high in the population analysed, especially in patients with pancreatic insufficiency [4,21].

ETI, in addition to having proven beneficial effects on respiratory function, sweat electrolyte levels, and general clinical conditions [51,54], is effective not only in improving nutritional status but also in leading to increased serum levels of fat-soluble vitamins.

## Figures and Tables

**Table 1:** Nutritional classification for cystic fibrosis patients.

	M	F
optimal	≥23	≥22
normal weight	20–23	20–22
underweight	18–20	18–20
malnutrition	<18	<18

**Table 2:** BMI z-scores classes in age group 2–18 years.

BMI z-Score:	69 Males N (%)	66 Females N (%)
≥0 SDS	38 (55.1%)	34 (51.5%)
<0 SDS ≥ −1 SDS	21 (30.4%)	21 (31.8%)
<−1 SDS ≥ −2 SDS	7 (10.1%)	9 (13.6%)
<−2 SDS	3 (4.3%)	2 (3.0%)

**Table 3:** Absolute values of BMI in adults.

BMI Class	109 Males N (%)	71 Females N (%)
≥23(M) o ≥22(F) optimal weight	47 (43.1%)	21 (29.6%)
20–23(M) o 20–22(F) normal weight	44 (40.4%)	27 (38.0%)
18–20 underweight	15 (13.8%)	18 (25.4%)
<18 malnutrition	3 (2.7%)	5 (7.0%)

**Table 4:** Serological levels of fat-soluble vitamins in patients on therapy with CFTR modulators.

	25(OH)D (ng/mL)			Vit A (mcg/dL)			Vit E (mcg/dL)		
	Basal	Post-Treatment	*p* Value	Basal	Post-Treatment	*p* Value	Basal	Post-Treatment	*p* Value
Iva N = 9	28.4 ± 9.5	36.7 ± 11.6	*p* = 0.12	44.7 ± 18.8	48.7 ± 7.6	*p* = 0.56	1337.8 ± 378.9	1762 ± 408.5	*p* = 0.036
LumIva N = 42	25.9 ± 11.5	24.9 ± 14.7	*p* = 0.73	36.4 ± 10.9	43.5 ± 13.1	*p* = 0.008	967.1 ± 282.7	938.8 ± 273.6	*p* = 0.64
ETI N = 29	23.5 ± 7.6	29.2 ± 9.5	*p* = 0.015	37.4 ± 13.0	47.4 ± 15.8	*p* = 0.011	1054.3 ± 208.0	1213.4 ± 219.3	*p* = 0.006

## Data Availability

The data presented in this study are available upon request from the corresponding author.

## Abbreviations

25(OH)D	25-hydroxyvitamin D
BMI	body mass index
CDC	Centers for Disease Control and Prevention
CF	cystic fibrosis
CFSPID	cystic fibrosis screening positive with inconclusive diagnosis
CFTR	CF transmembrane conductance regulator
ETI	Elexacaftor/Tezacaftor/Ivacaftor
F	female(s)
Iva	Ivacaftor
LumIva	Lumacaftor/Ivacaftor
M	male(s)
WHO	World Health Organization
